# Pair distribution functions of amorphous organic thin films from synchrotron X-ray scattering in transmission mode

**DOI:** 10.1107/S2052252517009344

**Published:** 2017-07-10

**Authors:** Chenyang Shi, Rattavut Teerakapibal, Lian Yu, Geoff G. Z. Zhang

**Affiliations:** a Drug Product Development, Research and Development, AbbVie Inc., 1 North Waukegan Road, North Chicago, IL 60064, USA; b School of Pharmacy, University of Wisconsin–Madison, Madison, WI 53705, USA

**Keywords:** pair distribution functions, amorphous organic thin films, transmission X-ray scattering, indomethacin

## Abstract

Using high-brilliance high-energy synchrotron X-ray radiation, for the first time the total scattering of a thin organic glass film deposited on a strongly scattering inorganic substrate has been measured in transmission mode. The organic thin film was composed of the weakly scattering pharmaceutical substance indomethacin in the amorphous state. The film was 130 µm thick atop a borosilicate glass substrate of equal thickness. The atomic pair distribution function derived from the thin-film measurement is in excellent agreement with that from bulk measurements. This ability to measure the total scattering of amorphous organic thin films in transmission will enable accurate *in situ* structural studies for a wide range of materials.

## Abstract 2

Using high-brilliance high-energy synchrotron X-ray radiation, for the first time a robust pair distribution function signal has been collected, in transmission mode, on a weakly scattering amorphous organic thin film deposited on a borosilicate glass substrate.

## 1. Introduction   

The lack of long-range order in liquids and glasses makes their structural characterization more difficult than for crystalline materials. Determination of the atomic pair distribution function (PDF) from total scattering has long been an important tool for characterizing amorphous materials and a foundation for developing their structural models (Narten & Habenschuss, 1984 ▸; Benmore *et al.*, 2011 ▸). The accurate determination of PDFs requires a Fourier transform of the ‘total’ scattering function, *i.e.* the scattering function that covers a sufficiently wide range of momentum transfer, typically measured with high-energy X-rays available from a synchrotron source in the transmission geometry.

Amorphous organic thin films are essential for many advanced technologies. Organic light emitting diodes (OLEDs), for example, consist of multiple layers formed by sequential physical vapor deposition. Determining the structure of these thin-film materials is required for optimizing their properties and performance. These thin-film materials, however, present great difficulties for the determination of their PDF because, in the transmission geometry, scattering from the substrate typically dominates the total signal. This has limited X-ray scattering studies to the reflection mode (Dawson *et al.*, 2012 ▸), while for transmission measurements the sample would have to be scraped off the substrate and accumulated before measurement, running the risk of destroying the original structure.

A solution to the problem of thin-film characterization is to take advantage of the highly penetrating X-ray beams available at third-generation synchrotron facilities. To this end, Jensen *et al.* (2015 ▸) reported the first measurement of a strongly scattering FeSb_3_ film 360 nm thick deposited on a 170 µm thick borosilicate glass substrate, and their result is consistent with that of Bauers *et al.* (2015 ▸) on the same film material that had been exfoliated and gathered in a Kapton tube. In this short communication, we extend this technique to weakly scattering organic glass films. We report the first thin-film PDF measurements on a pharma­ceutical substance, indomethacin (IMC, chemical formula C_19_H_16_ClNO_4_, mol­ecular weight 357.787 g mol^−1^) in the amorphous state. The structure function and PDFs extracted from thin-film measurements are in excellent agreement with those from bulk measurements. IMC is arguably the best studied amorphous pharmaceutical compound and an often-used model for molecular glasses (Bates *et al.*, 2006 ▸; Xiang & Anderson, 2013 ▸; Yuan *et al.*, 2015 ▸). The new capability developed here has the potential to advance the structural characterization of similar organic materials.

## 2. Experimental   

**Figure 1:**
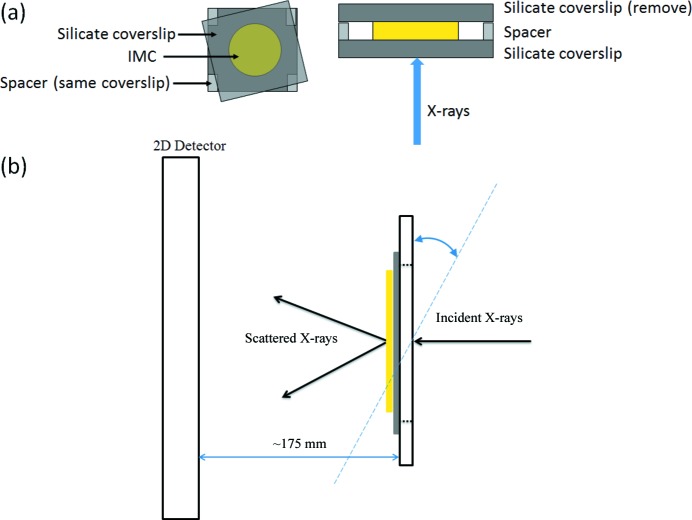
(*a*) Preparation of an IMC thin film. (*b*) Experimental setup for thin-film X-ray scattering measurements in transmission. The sample can be rotated if necessary.

The IMC powder sample was purchased from Alfar Aesar (lot number M19C065, purity >99%) and used without further purification. The as-received sample has the γ-form structure. As reported by Kistenmacher & Marsh (1972 ▸), the crystal structure is triclinic with space group 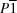 and cell constants *a* = 9.295 Å, *b* = 10.969 Å, *c* = 9.742 Å, α = 69.38°, β = 110.79° and γ = 92.78°, with *Z* = 2. A thin-film IMC sample was prepared by melting the crystalline powder between two silicate coverslips (Fig. 1*a* ▸). The distance between the coverslips was controlled using spacers (small pieces of the same coverslip) to be 130 µm. The assembly was cooled by contact with an aluminium block at room temperature to produce a glass film of IMC. The top coverslip was removed prior to the synchrotron X-ray experiments. The edges of the coverslip were taped to a custom-made aluminium frame with a square opening (2 × 2 cm), which was then loaded onto a goniometer head. In this work, the X-ray beam struck the film sample perpendicularly from the side of the coverslip (Fig. 1*b* ▸). The X-rays that penetrated the substrate were scattered by the IMC film and the scattered signal was detected by a two-dimensional detector. If necessary, the film can be rotated about an axis perpendicular to the X-ray beam, as shown with the two-headed arrow, to interrogate potential anisotropies. For conventional bulk PDF measurements, crystalline or amorphous IMC was loaded into Kapton capillary tubes with 0.0435 inch OD and 0.0395 inch ID (Cole–Parmer, EW-95820-09).

Synchrotron X-ray total scattering experiments were conducted on beamline 11-ID-B at the Advanced Photon Source, Argonne National Laboratory (Illinois, USA). All measurements were performed at room temperature. The rapid-acquisition pair distribution function (RaPDF) technique (Chupas *et al.*, 2003 ▸) was used with an X-ray energy of 58.705 keV (λ = 0.2112 Å). A Perkin–Elmer amorphous Si two-dimensional image-plate detector (2048 × 2048 pixels and 200 × 200 µm pixel size) was mounted orthogonal to the beam path with a sample-to-detector distance of ∼175 mm. The raw two-dimensional data were azimuthally integrated and converted to one-dimensional intensity *versus* 2θ using *FIT2D* (Hammersley *et al.*, 1996 ▸). *PDFgetX3* (Juhás *et al.*, 2013 ▸) was used to correct and normalize the diffraction data and to obtain the PDF, *G*(*r*), by Fourier transformation. A value of *Q*_max_ of 22.5 Å^−1^ was used for all samples.

## 3. Results and discussion   

**Figure 2:**
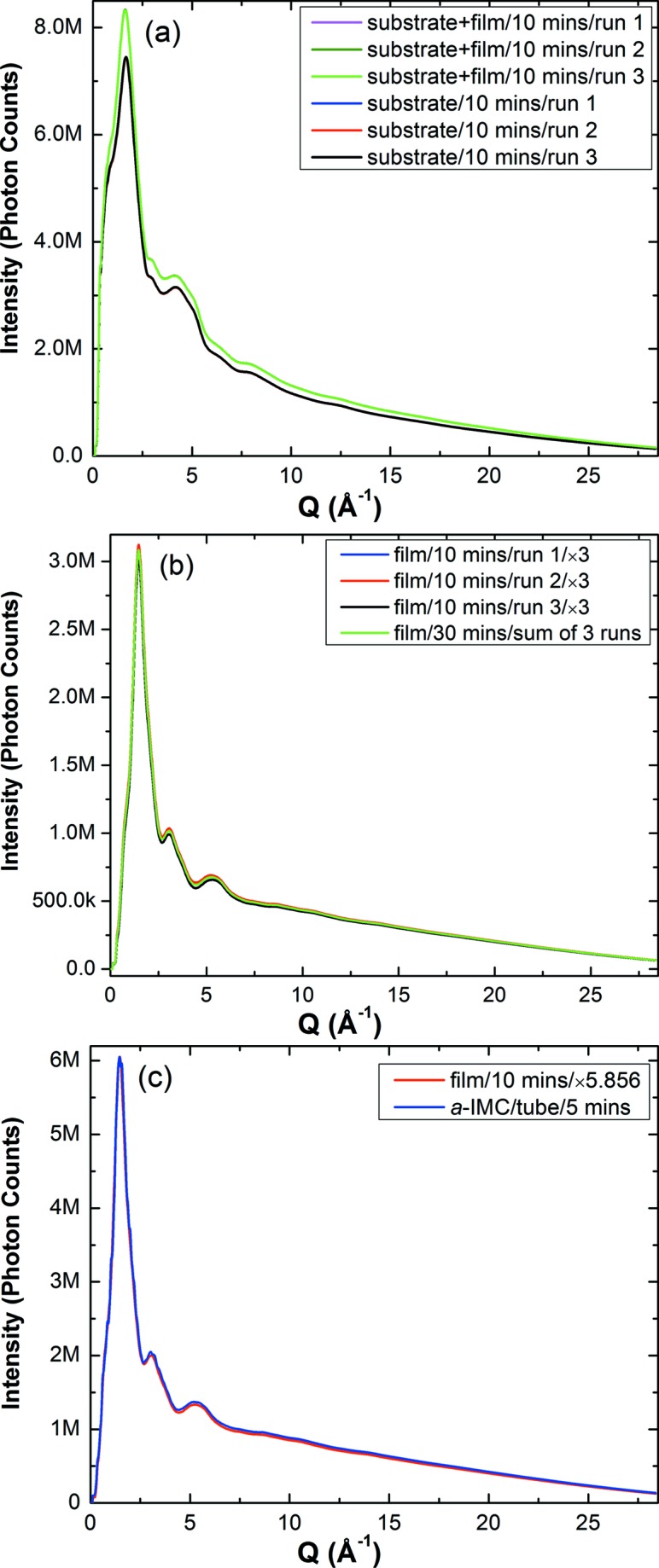
(*a*) Raw diffraction intensities for the thin-film sample including the substrate and for the substrate itself. (*b*) Diffraction intensities for the thin film only from four independent measurements. The 10 min runs were scaled to compare with the 30 min run. (*c*) The scattering signals for *a*-IMC from the tube (blue) and the thin-film (red) methods. The thin-film signal was scaled to match the signal from the tube method.

Fig. 2 ▸(*a*) shows the representative *I*(*Q*) data. Although the silicate substrate dominates the scattering intensity, the signal from the organic thin film can be accurately detected. The sample signal amounts to ∼11% of the total photon count at maximum intensity. Subtracting the background from the total signal yielded the signal from the sample film (Fig. 2 ▸ *b*). Notice the excellent agreement between different runs, all with a reasonable data-collection time of 10 min, and between results corresponding to different collection times (10 and 30 min). In fact, these results are indistinguishable within experimental error, indicating the robustness and feasibility of thin-film X-ray scattering measurements in transmission. Although the scattering intensity from an inorganic silicate substrate vanishes less quickly with increasing *Q* (*Q* is the momentum transfer, defined as *Q* = 4πsinθ/λ) compared with an organic Kapton tube, the background-subtracted scattering signals from both methods are indistinguishable (Fig. 2 ▸ *c*). This, again, demonstrates the robustness of the thin-film method.

**Figure 3:**
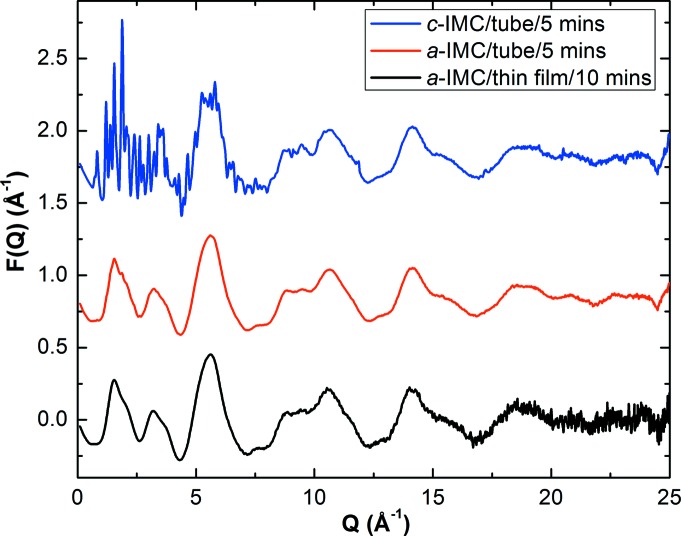
*F*(*Q*) for *a*-IMC obtained from thin-film and bulk measurements. For reference, the *F*(*Q*) for *c*-IMC (γ polymorph) is also shown.

The raw diffraction intensities were corrected for the background, Compton scattering and self-scattering, to obtain a coherent scattering intensity *I*_c_(*Q*). A structure function *S*(*Q*) is calculated *via* equation (1):
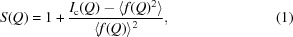
where 〈*f*(*Q*)〉 is the sum of the X-ray scattering form factors of all the atoms in IMC weighted by their concentrations, and 〈*f*(*Q*)^2^〉 is the sum of the squared X-ray scattering form factors of all the atoms in IMC weighted by their concentrations. A reduced structure function *F*(*Q*) is further calculated *via* equation (2):
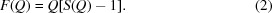
The PDF is obtained by a Fourier sine transform of *F*(*Q*) *via* equation (3): 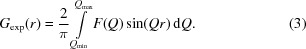
Experimental *F*(*Q*) data are shown in Fig. 3 ▸. Notice the excellent agreement between the *F*(*Q*) obtained from the thin-film sample and that from the bulk sample (measured in a Kapton tube using the same experimental setup). The bulk *F*(*Q*) has a higher signal-to-noise ratio in the high-*Q* range (>15 Å^−1^), a result of both a thicker sample in the beam path and lower scattering by the Kapton tube. Nevertheless, the thin-film samples still offer a large useable *Q* range up to 22.5 Å^−1^ for PDF calculations (see below).

The *F*(*Q*) of amorphous IMC (*a*-IMC) differs significantly from that of crystalline IMC (*c*-IMC, γ polymorph), as expected. The latter features sharp Bragg peaks at low *Q*. Notice, however, the agreement between the *F*(*Q*) functions of the crystalline and amorphous samples above *Q* ≃ 7 Å^−1^. Given that this region is dominated by intramolecular correlations, the agreement indicates that IMC has similar mol­ecular conformations in the crystalline and amorphous states.

**Figure 4:**
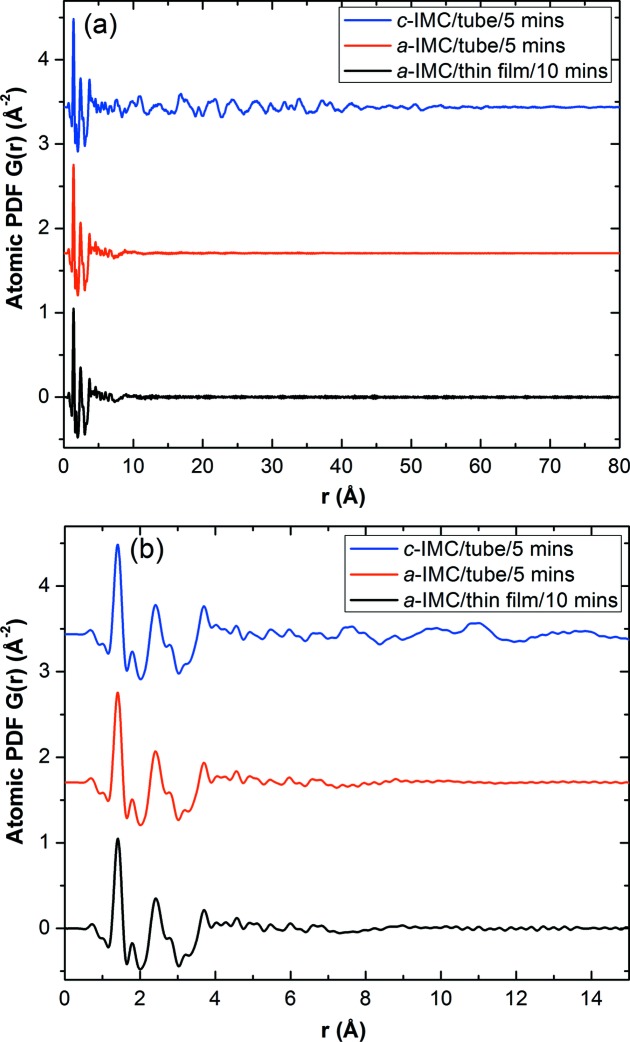
PDFs of crystalline and amorphous IMC plotted over *r* ranges of (*a*) up to 80 Å and (*b*) up to 20 Å. The measured crystalline PDF is also plotted as a reference.

Fig. 4 ▸ shows the PDF of amorphous IMC calculated from the thin-film *F*(*Q*). This calculation used a *Q*_min_ of 0.3 Å^−1^ and a *Q*_max_ of 22.5 Å^−1^ [see equation (3)]. We find that the PDF computed from the *F*(*Q*) of the thin-film sample is in excellent agreement with that from the *F*(*Q*) of the bulk sample, also shown in Fig. 4 ▸. To quantify the similarity between the two *a*-IMC PDFs, the Pearson product–moment correlation co­efficient is calculated, for which a value of 1 means a complete correlation and −1 anti-correlation. This coefficient is 0.9937 for the two curves from 0 to 80 Å. This agreement, again, validates the thin-film method for determining the PDF.

Fig. 4 ▸ also includes the PDF of crystalline IMC. The oscillations persist up to ∼80 Å, demonstrating the *Q* resolution limit at the current PDF experimental settings. The amorphous PDF signals, on the other hand, vanish at a much smaller distance (∼12 Å). It is evident that the amorphous and crystalline structures have very similar PDFs at short distances (<3.5 Å), as expected from their similar *F*(*Q*) functions at high *Q* values. This agreement indicates that the molecular structures in crystalline and amorphous IMC are similar.

To extract further structural details, one can adopt the methodology of Narten & Habenschuss (1984 ▸) and Prill *et al.* (2015 ▸), where the total PDF is decomposed into its intra- and intermolecular components. For an amorphous organic material, reverse Monte Carlo and molecular dynamics simulations are promising techniques. Modeling efforts on *c*-IMC and *a*-IMC are currently ongoing.

## 4. Conclusions   

In summary, this work has demonstrated that, by taking advantage of high-brilliance high-energy synchrotron X-ray radiation, it is possible to measure the total scattering of a thin organic glass film supported on a strongly scattering substrate in the transmission mode and thus determine its atomic pair distribution function. For an indomethacin (IMC) film 130 µm thick on a borosilicate glass substrate of equal thickness, a 10 min measurement time is sufficient. The thin-film PDF is in excellent agreement with the PDF obtained for a bulk sample. With minimal optimization of the current procedure and longer integration, we expect that total scattering measurements will be possible in transmission mode for supported weakly scattering organic thin films that are less than 1 µm thick. This ability will enable structural studies of a wide range of organic materials prepared as supported films, including ultra-stable organic glasses prepared by physical vapor deposition (Swallen *et al.*, 2007 ▸) and epitaxially grown thin films.

## 5. Disclosure   

AbbVie and the University of Wisconsin–Madison participated jointly in study design, research, data collection, analysis and interpretation of data, and writing, reviewing and approving the publication. R. Teerakapibal is a graduate student at the University of Wisconsin–Madison; L. Yu is a professor at the University of Wisconsin–Madison. They have no additional conflicts of interest to report. C. Shi and G. G. Z. Zhang are employees of AbbVie and may own AbbVie stock.
